# Mental health mobile application self‐help for adolescents exhibiting psychological distress: A single case experimental design

**DOI:** 10.1111/papt.12436

**Published:** 2022-11-07

**Authors:** Kiran Badesha, Sarah Wilde, David L. Dawson

**Affiliations:** ^1^ Clinical Psychology University of Lincoln Lincoln UK

**Keywords:** adolescent, CBT apps, mHealth, mobile apps, psychological distress, self‐help

## Abstract

**Objectives:**

The present demand for child and adolescent mental health services exceeds the capacity for service provision. Greater research is required to understand the utility of accessible self‐help interventions, such as mobile apps. This study sought to investigate whether use of a mental health app, underpinned by CBT, led to changes in psychological distress amongst adolescents. Mechanisms of change were examined, specifically whether changes are attributable to cognitive strategies.

**Design:**

This study utilised a multiple‐baseline single‐case experimental design, tracking variables across baseline and intervention phases. Surveys assessing participant experience were also administered.

**Methods:**

Five participants with moderate‐to‐severe levels of psychological distress engaged with a CBT‐based app over five weeks. Participants were recruited from both a well‐being service and the general population. Supplementary weekly calls to participants offered clarification of app content.

**Results:**

A small overall effect of the intervention of psychological distress was evident; however, outcomes were dependent on the analysis conducted. The intervention appeared to promote an increase in use of adaptive cognitive strategies but not negative thinking styles. The CBT app did not promote changes in participant well‐being. Participant feedback highlighted practical challenges of utilising the app.

**Conclusions:**

The clinical benefits of app‐based CBT were small, and a range of barriers to engagement were recognised. While further research is required, caution should be exercised in the interpretation of studies reporting on app effectiveness.

**Practitioner Points**

- Use of the CBT‐based app led to small improvements in psychological distress across three of five participants.
- Changes in distress appeared temporally related to adaptive cognitive strategies, but not to maladaptive cognitive styles.
- Use of the CBT‐based app did not lead to any reliable or clinically significant changes in well‐being.
- The CBT‐based app was considered by participants to be demanding on time and motivation.

## BACKGROUND

As the concept of ‘psychological distress’ is broadly defined, the statistics of distress amongst young people are often not reported and instead rely on diagnostic classifications (Merikangas et al., 2009). An estimated 16% of young people in the UK fulfil a probable mental health diagnosis (NHS Digital, 2020). As these rates have increased by over 5% in three years (NHS Digital, 2020), an increase in psychological distress would be expected. Such increase in distress is an important public health challenge, given the possible long‐lasting effects of poor mental health (Belfer, 2008; Kieling et al., 2011; Veldman et al., 2015).

At present, a Children and Young People's Improving Access to Psychological Therapies (CYP‐IAPT) model is utilised within England; this stepped‐care approach proposes to cost less clinician time and enable faster treatment to young people (Yeguez et al., 2020), including those with mild‐to‐moderate distress. Low‐intensity interventions have demonstrated various successes across settings (Cobham et al., 2017; Creswell et al., 2017; Michelson et al., 2020; Rickwood et al., 2015). However, despite efforts to enhance accessibility to services, there remains limited capacity to accept referrals, with approximately 132,700 young people rejected from child and adolescent mental health services (CAMHS) in England (Crenna‐Jennings & Hutchinson, 2020). There also remain long waiting times nationally, with a third of referred young people waiting longer than 18 weeks in Scotland (Public Health Scotland, 2022), and a quarter waiting longer than this period within Northern Ireland (British Medical Association, 2017). Such wait times pose an additional barrier to accessing support (Punton et al., 2022).

Given the disparity between the need for and availability of support, there is growing recognition by Public Health England (2021) of the requirement for young people's well‐being to be prioritised. It is further noted that large proportions of adolescents do not seek support for their psychological difficulties for various reasons (Gulliver et al., 2010; Plaistow et al., 2014). Given the number of young people requiring support, widely accessible, flexible and cost‐effective resources to reduce distress should be made available.

Self‐help interventions utilising evidence‐based therapy models have demonstrated efficacy for various psychological difficulties (Bennett et al., 2019). In recent years, advancements in technology have enabled self‐help to be extended to internet‐based interventions (Grist et al., 2019), increasing the accessibility of self‐help resources. Digital health interventions, including computerised cognitive behavioural therapy (cCBT), have demonstrated effectiveness in reducing distress amongst adolescents. For example, a meta‐analysis of studies recruiting 11‐19‐year‐old participants demonstrated small but significant effects of cCBT on depression and anxiety symptoms, with comparable outcomes to face‐to‐face interventions (Wickersham et al., 2022), demonstrating the value of such interventions.

Despite the potential benefits of digital interventions, there remain barriers, such as low uptake and engagement (Bevan Jones et al., 2022) and limited understanding regarding the active components of the interventions (Hollis et al., 2017). One type of digital intervention attracting greater attention over recent years includes mobile apps. Thus far over 165,000 mental health apps exist (IMS Institute for Healthcare Informatics, 2015); this has likely grown over recent years. While over 80% of 12‐15 year olds in the UK are reported to own a smartphone (Ofcom, 2019), it is suggested that apps could offer young people more timely and cost‐effective support and could assist in overcoming obstacles such as help‐seeking stigma (Koh et al., 2022). Nonetheless, challenges also exist with apps, including barriers to engagement, limited crisis support and lack of evidence‐based approaches (Koh et al., 2022). Furthermore, some apps may fail to adhere to clinical guidelines (Torous & Roberts, 2017), highlighting potential for iatrogenic effects (Lui et al., 2017).

While the literature investigating apps demonstrates some potential for positive psychological outcomes amongst young people, much of the research investigating app effectiveness across young people includes inconsistent operationalisation of terms ‘young adult’ and ‘adolescent’ (Leech et al., 2021). Thus, research solely exploring apps across adolescent samples remains limited (Grist et al., 2017; Leech et al., 2021). This limited evidence is surprising given the national drivers promoting digital interventions, including ‘quality assured apps’, including National Institute for Health and Care Excellence (NICE, 2019) guidelines and the Department of Health (2012) Future in Mind initiative. Nonetheless, while the COVID‐19 pandemic has highlighted the role for digital interventions (Moreno et al., 2020), the use and recommendation of mobile apps remain under scrutiny (Skorburg & Yam, 2022; Torous, Myrick et al., 2020; Whelan et al., 2020).

Despite policies promoting app technologies, there is limited understanding around whether apps are an effective resource, and if so, through which mechanisms change might occur (Badesha et al., 2022; Hollis et al., 2017). Active ingredients underpinned by psychotherapeutic models have yet to be explicitly investigated within app interventions. Despite a preponderance of apps utilising principles of CBT (Badesha et al., 2022), it is not understood whether improvements in clinical symptomatology occur through processes aligned with this model. Within face‐to‐face CBT interventions, cognitive mechanisms, for example changes in cognitive distortions (Drapeau et al., 2019; Shirk et al., 2013) and negative automatic thoughts (Kaufman et al., 2005), are suggested to reduce depressive symptoms. It may be predicted that change might occur through similar mechanisms when CBT is adapted for apps. Additionally, apps utilising alternative psychotherapeutic models such as mindfulness would be expected to promote change via mechanisms including self‐regulation, or cognitive, emotional and behavioural flexibility (Shapiro et al., 2006). Furthermore a ‘digital placebo effect’ is also possible (Torous & Firth, 2016). Findings from meta‐analyses report smaller effect sizes when app conditions were compared with active rather than non‐active control conditions (Firth, Torous, Nicholas, Carney, Pratap, et al., 2017; Firth, Torous, Nicholas, Carney, Rosenbaum, & Sarris, 2017), suggesting change may not be wholly due to psychotherapeutic ingredients of app interventions.

### Rationale for study

While internet‐based interventions have demonstrated their value in offering accessible self‐guided support, a growing number of apps are becoming available, increasing accessibility to self‐help tools. Research to investigate mental health mobile apps is needed to understand whether they contribute to changes in psychological distress amongst adolescents. This is particularly pertinent, given the increasing need for accessible means of support. While there is a rationale for recommending apps, there are currently various issues in doing so given that the utility of apps is under‐researched amongst younger age groups. Further research is required to understand the external validity, namely the applicability (Murad et al., 2018), of app‐interventions in practice. Such investigation would be valuable for primary care, mental health services, education providers and other services, to understand the real‐world application of a CBT app for young people.

### Aims and objectives

This study aims to investigate changes in psychological distress amongst adolescents while using a mental health app. To address this aim, evidence of trend changes across phases is to be analysed, as well as evidence of reliable and clinically significant change in psychological distress across cases. Secondly, this study seeks to investigate processes of change across phases, by inspecting changes in attribution styles and analysing survey responses of participants. Finally, this study aims to understand barriers and facilitators of a mental health app by investigating patterns of app usage and by evaluating feedback from participants.

## METHOD

This research was approved by a Research Ethics Committee (REC), the NHS Health Research Authority (HRA), and a University Ethics Committee. The study complied with the British Psychological Society code of human research ethics.

### Design

This study utilised a mixed methods single‐case series using a non‐concurrent multiple baseline design. Single‐case experimental designs (SCEDs) are used as a prospective investigation of manipulated variables, with regular repetition of measures across baseline and intervention phases, tracking variables across time. Within multiple‐baseline designs, participants complete baseline and intervention phases with staggered introduction of the intervention phase across participants (Christ, 2007); this can offer greater experimental control where learning cannot be reversed (Baer et al., 1968, 1987). A mixed methods design was adopted, to assess open‐ended survey responses by participants following the intervention. Including a feedback component was proposed to further develop an understanding of the quantitative results (Bryman, 2006) and to offer greater applicability (Murad et al., 2018).

### Recruitment

Individuals aged 13–18 years were eligible to participate in the study if they had capacity to provide informed consent and were experiencing psychological distress. For those aged below 16 years, additional informed consent from legal parents/guardians was required; adolescents aged 13–15 years would be excluded if parental consent was not provided. Individuals were excluded if accessing crisis support, to ensure necessary support was not withheld. Exclusions applied to those unable to speak or understand English, as the app and measures were only available in English, and in cases where smart devices and internet could not be accessed.

A minimum of three participants is proposed to represent three replications of effect within SCEDs (Lane & Gast, 2014); thus, recruitment of between three and six participants to completion was proposed. To achieve this sample size, two recruitment strategies were utilised.

#### Service recruitment

Participants were recruited from an NHS well‐being service for young people. Limited details pertaining to the service are offered, to ensure confidentiality and anonymity (Tate et al., 2016). First contact was carried out with a Mental Health Practitioner; young people meeting relevant criteria and registering an interest in participation as part of their care were sent information leaflets and consent forms. The researcher contacted them, providing further information about the study, prior to recruitment. Consent forms were signed and returned to the service using prepaid envelopes.

#### Community recruitment

Recruitment advertisements directed towards young people were disseminated via social media platforms Facebook and Instagram. Adverts directed prospective participants to a Qualtrics survey, to express an interest and provide contact emails. Information leaflets were sent via email to those expressing an interest. Those wishing to proceed completed electronic consent forms; these were also completed by parents/carers of those under 16.

### Participants

Eleven participants were recruited across both recruitment strategies, with a total of five participants completing the study (Table 1). The preponderance of females within the sample is consistent with the gender imbalance demonstrated within adolescent willingness to access mental health services (Chandra & Minkovitz, 2006). None of the participants had previous specialist support for their well‐being.

**TABLE 1:** Demographics of participants completing the study

Participant	Gender	Age	Ethnicity	Duration of distress
1	Female	15	White British	6–10 years
2	Female	17	Pakistani British	1–2 years
3	Female	16	Bangladeshi British	6–10 years
4	Female	17	Pakistani British	1–2 years
5	Non‐binary	16	White British	3–5 years

### Measures

Measures were selected to assess psychological outcomes (distress and well‐being), along with processes consistent with the therapy models utilised within the app (Table 2).

**TABLE 2:** Summary of measures

Measure	Use	Psychometric properties	Example Items
Kessler‐10 (K10; Kessler et al., 2002)	10 items to assess psychological distress Assessed: three times weekly	Good reliability and predictive validity *ω* = 0.97 (Smout, 2019)	‘During the last week, how often did you feel that everything was an effort?’
Warwick‐Edinburgh Mental Wellbeing Scale (WEMWBS; Tennant et al., 2007).	14 items to assess well‐being Assessed: pre‐, mid‐, post‐intervention	Good internal consistency *α* = 0.87(Clarke et al., 2011)	‘I've been feeling optimistic about the future’
Cognitive Emotion Regulation Questionnaire – short version (CERQ‐Short; Garnefski & Kraaij, 2006)	18 items to assess cognitive strategies; adaptive cognitive strategies (8 items) negative strategies (10 items) Assessed: three times weekly	Good internal consistency Subscale range: *α* = 0.68 to *α* = 0.81	Adaptive: ‘I think I can learn something from the situation’ Negative: ‘I keep thinking about how terrible it is what I have experienced’
CompACT‐8 (Morris et al., 2019)	8 item measure to assess behavioural awareness (BA), valued action (VA), openness to experience (OE), and psychological flexibility Assessed: pre‐, mid‐, post‐intervention	Acceptable internal consistency *α* = > 0.70 (Morris et al., 2019)	OE: ‘I work hard to keep out upsetting feelings’ BA: ‘I rush through meaningful activities without being really attentive to them’ VA: ‘I act in ways that are consistent with how I wish to live my life’
Change Survey	Survey based on nine key items (Elliott et al., 2001) Assessed: post‐intervention	N/A	‘What changes have you noticed in yourself?’

### Procedure

#### Pre‐intervention and baseline period

Once recruited, participants completed the measures online via the Qualtrics platform. Three baseline durations were identified a priori, to increase experimental control (Barlow et al., 2009; Kazdin, 2011; Kratochwill et al., 2010). Participants completed the baseline phase for two, three or 4 weeks. Allocation of baseline durations was not randomised but was instead based on order of consent form completion, as such, the first two participants completing consent forms were allocated a two‐week baseline period, the third and fourth completing the forms were allocated a three‐week baseline period and so on.

#### Intervention period

Participants were provided with the app to use on their own device and guided to complete the modules over 5 weeks. Participants were advised to complete one activity per day. Participants received weekly telephone or video calls from their practitioner or the lead researcher, depending on mode of recruitment. Calls lasted up to 30 minutes and served to provide a rationale for the content and to answer any questions. Upon completion of the intervention period, participants were invited to complete a change survey, an online form comprising a range of open questions regarding their experience.

### Intervention

Participants were provided with the app ‘Sanvello’, which was chosen from a selection of apps aimed to promote well‐being and mental health. This app has demonstrated effectiveness (Moberg et al., 2019) and its content (Table 3) adhered to CBT principles. The process of app selection involved a task group comprising CAMHS staff and peer support workers. ‘Sanvello’ met criteria pertaining to evidence of effectiveness, security, privacy, and confidentiality, in line with UK standards (Public Health England, 2017). App accounts were created and managed by the first author, to ensure participants' personal data was not utilised.

**TABLE 3:** Intervention modules

Guided modules	Timeframe	Content
Feeling better	Days 1–7	Introduction to CBT
Taking control	Days 8–18	CBT: Psychoeducation, setting goals, identifying negative thoughts, challenging thoughts
Building confidence	Days 19–28	CBT: Reframing and reappraisal
Mindfulness	Days 29–35	Mindfulness: Psychoeducation, skills building, practice, mindfulness of emotion and thoughts, nurturing compassion

### Analysis

Change was analysed according to the What Works Clearinghouse (WWC) guidance (Kratochwill et al., 2010). As visual analysis remains the dominant evaluation method used within SCEDs (Kazdin, 2021), graphs for each participant were inspected for trend changes between baseline and intervention phases. Stability was assessed according to Lane & Gast (2014) criteria of up to 25% variation. The primary method of analysis was visual analysis of overlap in scores of psychological distress, utilising both dual criterion (DC) and percentages exceeding the median (PEM) methods.

Given that different criteria used to conduct visual analysis may lead to different conclusions (Kazdin, 2021), additional analyses in line with relevant guidance (Kratochwill et al., 2010; Lane & Gast, 2014; Wolery & Harris, 1982) were proposed (Table 4). Reliable change and clinically significant change scores were determined using the method proposed by Jacobson and Truax (1991), utilising normative data for adolescents for the K10 (Andrews & Slade, 2001) and WEMWBS (Clarke et al., 2011).

**TABLE 4:** Analysis plan

Method of analysis	Rationale	Aims Assessed	Limitations and proposed solutions
Visual analysis	Provides a graphical presentation of data between phases and between participants.	Aims 1, 2	Variety of methods available to inspect data, which can lead to variance in conclusions drawn. Supplemented by calculations of stability, level, trend, overlap.
Stability envelope calculations	To assess variability in each phase and identify threats to internal validity.	Aim 1, 2	Stability not always achieved. Use of multiple baseline design and Tau‐U analysis.
Trend: Split‐middle method	To assess trend stability and estimate trend direction within each condition.	Aims 1, 2	Potential for Type 1 errors. Supplemented by PEM and DC methods.
Overlap: Dual criterion (DC) method and Percentages exceeding the median (PEM)	Primary analysis, combining two conventions to test for phase differences of non‐overlap.	Aims 1, 2	PEM alone lacks sensitivity to magnitude of data points exceeding median. Supplemented by DC method.
Reliable change index (RCI)	Assessment of statistically reliable change or change due to chance or variability.	Aim 1	Dependent on available normative data.
Clinically significant change (CSC)	Assessment of change according to clinical and non‐clinical levels of outcomes.	Aim 1	Dependent on available normative data. May not account for client‐centred meaningful change. Considered in conjunction with survey feedback.
Tau‐U analysis	Analysis of phase differences and assessment of overall effect size, with corrected baselines trends.	Aims 1, 2	Vague and inconsistent terminology, with may lead to difficulties interpreting results. Supplemented by PEM, DC, and visual analysis.
Framework analysis	Analysis of written responses to open‐ended questions pertaining to participant views, allowing for within‐participant and between‐participant analysis.	Aims 2, 3	Subjectivity of analysis. Utilising standardised guidance to conduct.

To analyse feedback provided within change surveys, the Framework method was adopted. The Framework method refers to a rigorous method of analysing qualitative data and has been used widely across health service research (Ritchie et al., 2013). Framework analysis is a step‐by‐step iterative process and can be used across various research questions, including those with an evaluative function (Gale et al., 2013; Ritchie & Spencer, 1994).

## RESULTS

### Participant attrition and adherence

#### Attrition

Throughout various stages of the study, six participants withdrew or were withdrawn (Table 5). Participants 6, 7 and 9 were discharged from the service without additional referrals, suggesting disengagement from services or perceived improvements. P1 and P8 were referred to CAMHS for specialist mental health support; for P1, this referral occurred during the last week of the intervention. In addition to this, P11 sought out additional psychotherapeutic input.

**TABLE 5:** Participant attrition

Participant	Age	Gender	Stage of dropout	Recruitment method	Reason for dropout
6	13	Female	Week 1	Service	Lack of motivation
7	17	Male	Week 2	Service	Lack of motivation
8	14	Female	Week 1	Service	Referral to crisis (discharged)
9	15	Non‐binary	End‐baseline	Service	Not suitable for service (discharged)
10	16	Female	Mid‐baseline	Non‐Service	Lack of time to participate
11	16	Female	End‐baseline	Non‐Service	Participation alongside exams increased stress

#### Adherence

All participants completing the study completed all CBT aspects of the intervention. P5 did not complete 50% of the final mindfulness‐based module. Patterns of module completion were monitored (Table 6). Despite recommendations to complete one activity per day, this was only demonstrated by P1, and occasionally P2. Participants 3, 4 and 5 appeared to complete much of the content towards the latter half of the week. A large proportion was completed on the days prior to, day of, or days following weekly calls.

**TABLE 6:** Patterns of module completion per week

Participant	Day one (%)	Day two (%)	Day three (%)	Day four (%)	Day five (%)	Day six (%)	Day seven (%)	Following week (%)
Week 1								
P1	14.29	14.29	14.29	14.29	14.29	14.29	14.29	
P2	14.29	‐	42.86	14.29	14.29	‐	14.29	‐
P3	14.29	‐	‐	‐	42.86	‐	42.86	‐
P4	28.57	‐	‐	‐	‐	‐	71.43	‐
P5	28.57	‐	‐	‐	‐	‐	14.29	57.14
Week 2								
P1	14.29	14.29	14.29	14.29	14.29	14.29	14.29	
P2	‐	‐	42.86	42.86	‐	‐	14.29	‐
P3	‐	‐	28.57	42.86	14.29	‐	‐	14.29
P4	‐	‐	‐	‐	28.57	‐	71.43	‐
P5	‐	‐	‐	‐	57.14	‐	28.57	14.29
Week 3								
P1	14.29	14.29	14.29	14.29	14.29	14.29	14.29	
P2	‐	‐	‐	42.86	14.29	‐	42.86	‐
P3	14.29	‐	‐	‐	85.71	‐	‐	‐
P4	‐	‐	‐	‐	14.29	‐	85.71	‐
P5	‐	‐	‐	57.14	‐	‐	42.86	‐
Week 4								
P1	14.29	14.29	14.29	14.29	14.29	14.29	14.29	
P2	‐	‐	14.29	14.29	14.29	‐	42.86	14.29
P3	‐	‐	‐	‐	100	‐	‐	‐
P4	‐	‐	‐	‐	‐	‐	71.43	28.57
P5	‐	‐	‐	‐	42.86	‐	57.14	‐
Week 5								
P1	14.29	14.29	14.29	14.29	14.29	14.29	14.29	
P2	33.33	‐	‐	‐	16.67	16.67	33.33	‐
P3	‐	‐	‐	‐	100.00	‐	‐	‐
P4	‐	‐	‐	‐	100.00	‐	‐	‐
P5	‐	‐	‐	‐	50.00	‐	‐	‐

*Note:* Shading denotes the day of weekly check‐in.

### Psychological outcomes

#### Psychological distress

Baseline stability is demonstrated in Table 7. Four participants (P1, P2, P3 and P5) demonstrated a stability envelope within 25% of the median value. P4 demonstrated baseline variability, with a 35% stability envelope. However, the baseline period was not extended for P4. During the intervention phase, all participants demonstrated acceptable levels of stability.

**TABLE 7:** Stability for psychological distress scores

Participant	Mean	Median	Range	Stability met (%)	Acceptability
Baseline					
1	47.17	47.00	44–50	0–10	Acceptable
2	27.50	27.00	27–32	0–10	Acceptable
3	32.78	33.00	28–36	0–10	Acceptable
4	18.78	18.00	14–26	> 25	Questionable
5	27.83	27.50	23–35	10–20	Acceptable
Intervention					
1	49.89	50.00	48–50	0–10	Acceptable
2	22.38	23.00	19–26	10–20	Acceptable
3	28.28	28.00	24–34	10–20	Acceptable
4	15.39	14.00	14–20	20–25	Acceptable
5	25.56	27.00	14–33	20–25	Acceptable

**FIGURE 1:**
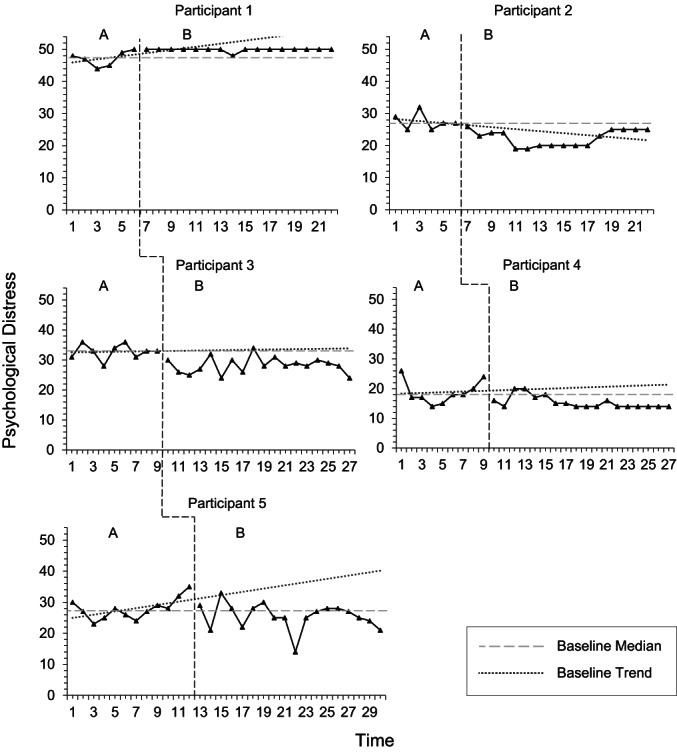
Psychological distress during baseline (A) and intervention (B)

Visual analysis of psychological distress did not demonstrate replication of effects across all participants (Figure 1). Upon assessment of trends using the split‐middle method, an upward trend in psychological distress was evident across all participants during baseline phases. However, during intervention phase, a downward trend was only evident for P5. When using PEM and DC methods to assess the effects of the intervention, change amongst all participants except P1 was found to be effective in reducing distress. When assessing change in pre‐ and post‐intervention scores using RCI and CSC methods of analysis, the change was reliable and clinically significant for three participants (Table 8).

**TABLE 8:** Changes in psychological distress scores

Total scores		Pre‐post analysis		
Participant	Pre	Post	Reliable change	Movement across threshold	PEM / DC
1	48	50	No change	No movement	0.00
2	29	25	No change	No movement	0.94
3	31	24	Improvement^a^	Severe to Moderate	0.94
4	26	14	Improvement^a^	Moderate to Likely Well	0.83
5	30	21	Improvement^a^	Severe to Mild	0.50

^a^ Clinically Significant Change using Criterion C.

Results are interpreted with caution, as assessment of reliable and clinically significant change using the median baseline value and post‐intervention scores demonstrated reliable improvement and clinically significant change in P3 only. Upon inspection of Figure 1, P2's scores of psychological distress appeared to increase towards the latter stages of the intervention stage, regressing towards the baseline median value. Inspection of P4's scores also demonstrate that the initial K10 score was greater than the median of baseline scores. As illustrated in Table 8, analysis of phase differences according to the PEM and DC methods shows that three participants (P2, P3 and P4) demonstrated treatment effects. Whereas P1 demonstrated no change, and P5 demonstrated no treatment effect.

Tau‐U statistics were calculated by correcting for influence of baseline trend for Participants 1, 2 and 3. Tau‐U calculations suggested that three participants exhibited a significant decrease in psychological distress, while P1 demonstrated a significant increase in this variable (Table 9). However, this increase was evident prior to commencement of intervention phase. In line with Vannest and Ninci's (2015) effect size categorisations, the phase differences demonstrated by P2 and P3 were large, while P4 demonstrated a moderate‐to‐large phase difference. However, these effect size estimates are to be interpreted relative to the relevant context. An overall weighted average for Tau‐U effect size calculation demonstrated significant but small phase differences.

**TABLE 9:** Summary of tau‐U Analysis comparing baseline and intervention phases across psychological distress scores

Participant	S	Tau‐U	*SD* Tau	*z* value	*p* value	90% CI
1	71	0.74	0.28	2.62	.0089**	[0.275, 1.00]
2	‐84	−0.88	0.28	−3.10	.002**	[−1.00, −0.410]
3	−125	−0.77	0.24	−3.21	.0013**	[−1.00, −0.377]
4	−106	−0.65	0.24	−2.73	.0064**	[−1.00, −0.259]
5	−85	−0.39	0.22	−1.80	.072	[−0.753, −0.034]

Weighted average	Tau‐U	Var‐Tau	*z* value	*p* value	95% CI
	−0.41	0.11	−3.62	.0003**	[−0.6340, −0.1886]

*Note*: SD = standard deviation; CI = confidence interval; S statistic calculated by subtracting number of negative matrix values from number of positive matrix values.

*p < .05, **p < .01.

#### Well‐being

**FIGURE 2:**
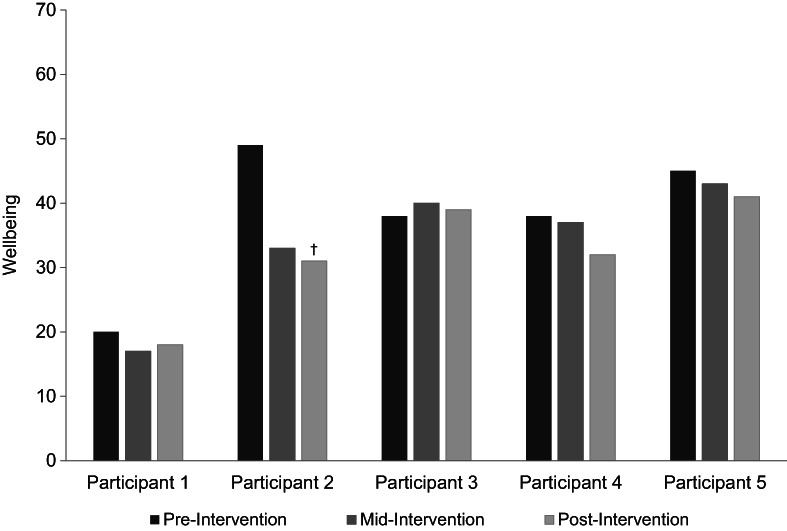
Scores of well‐being at baseline, mid‐intervention and post‐intervention phases

Well‐being did not increase during the intervention phase. Four participants exhibited a potential decrease in well‐being over time (Figure 2), with one participant demonstrating a reliable deterioration between pre‐ and post‐intervention phases. This is consistent with P2's scores of psychological distress, which increased towards the latter stages of the intervention phase. Participants' well‐being scores were indicative of low levels of well‐being at all time points, with the exception of P2 and P5, who demonstrated ‘average’ well‐being at the pre‐intervention stage (Gremigni & Bianco, 2012).

### Processes of change

#### Cognitive emotion regulation

All participants demonstrated a stability envelope within 25% of the median value, for all cognitive strategies during baseline (Table 10). This was evident during the intervention phase, except for P1, whose stability envelope was 35% for adaptive strategies.

**TABLE 10:** Stability for cognitive regulation scores

Participant	Adaptive Strategies					Negative Strategies				
	Mean	Median	Range	Stability (%)	Acceptability	Mean	Median	Range	Stability (%)	Acceptability
Baseline										
1	15.00	15.00	15	0–10	Acceptable	29.00	28.00	27–34	10–20	Acceptable
2	25.83	25.50	25–29	0–10	Acceptable	34.83	35.00	33–36	0–10	Acceptable
3	22.56	22.00	19–27	10–20	Acceptable	36.89	37.00	33–41	10–20	Acceptable
4	22.56	22.00	20–26	10–20	Acceptable	31.33	32.00	28–34	0–10	Acceptable
5	16.33	16.00	12–22	20–25	Acceptable	26.42	26.00	21–33	10–20	Acceptable
Intervention										
1	21.35	22.00	12–29	>25	Questionable	32.41	34.00	15–37	0–10	Acceptable
2	28.94	30.00	18–32	10–20	Acceptable	35.00	35.00	33–38	10–20	Acceptable
3	27.47	29.00	22–31	10–20	Acceptable	33.00	34.00	29–39	10–20	Acceptable
4	20.47	20.00	20–25	0–10	Acceptable	32.00	32.00	31–33	0–10	Acceptable
5	19.11	19.00	17–22	10–20	Acceptable	26.06	26.50	22–29	10–20	Acceptable

**FIGURE 3:**
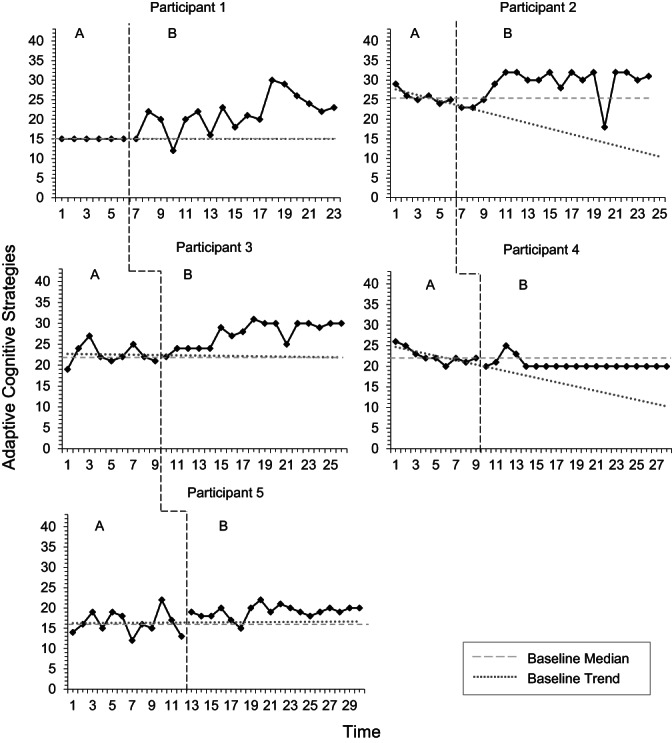
Use of adaptive cognitive strategies across baseline (A) and intervention (B) phases

Visual analysis of adaptive cognitive strategies (Figure 3) demonstrated replication of effects across three participants on all methods of analysis excluding split‐middle analysis. According to the split‐middle method, a change in trend between baseline and intervention phases was evident for Participants 1, 3 and 5. Analysis using PEM and DC methods demonstrated an increase in adaptive cognitive strategies during the intervention phase across four participants (P1, P2, P3 and P5; Table 11).

**TABLE 11:** Summary of analyses comparing cognitive strategies across baseline and intervention phases

Participant	Adaptive strategies							Negative strategies						
	PEM / DC	S	Tau‐U	*SD* Tau	*z* value	*p* value	90% CI	PEM / DC	S	Tau‐U	*SD* Tau	*z* value	*p* value	90% CI
1	0.88	84	0.84	0.28	2.94	.0033**	[0.363, 1.00]	0.06	71	0.70	0.28	2.49	.0129**	[0.235, 1.00]
2	0.78	70	0.65	0.28	2.33	.0196*	[0.191, 1.00]	0.00	1	0.01	0.28	0.03	.9734	[−0.447, 0.466]
3	0.94	120	0.78	0.24	3.23	.0012**	[0.385, 1.00]	0.24	−84	−0.55	0.24	−2.26	.0236*	[−0.948, −0.150]
4	0.11	−102	−0.60	0.24	−2.51	.0121**	[−0.988, 0.205]	0.16	11	0.06	0.24	0.27	.7867	[−0.327, 0.455]
5	0.94	135	0.63	0.22	2.86	.0043**	[0.265, 0.985]	0.35	−19	−0.09	0.22	−0.40	.6876	[−0.448, 0.272]

Weighted average	Tau‐U	Var‐Tau	*z* value	*p* value	95% CI	Weighted average	Tau‐U	Var‐Tau	*z* value	*p* value	95% CI
	0.44	0.11	3.91	.0001**	[0.220, 0.663]		0.01	0.11	0.05	.9565	[−0.2152, 0.2275]

*p < .05, **p < .01.

Tau‐U indices were calculated, correcting for baselines of P2 and P4. Tau‐U calculations suggested that four participants demonstrated significant increase in adaptive strategies between baseline and intervention phases. Participants 1, 3 and 5 demonstrated very large phase differences, while P2 demonstrated large phase differences. One participant (P4) demonstrated a significant decrease in adaptive strategies between phases but only a small effect. An overall weighted average Tau‐U calculation suggested a significant positive phase difference in adaptive strategies across participants, with a moderate effect size.

**FIGURE 4:**
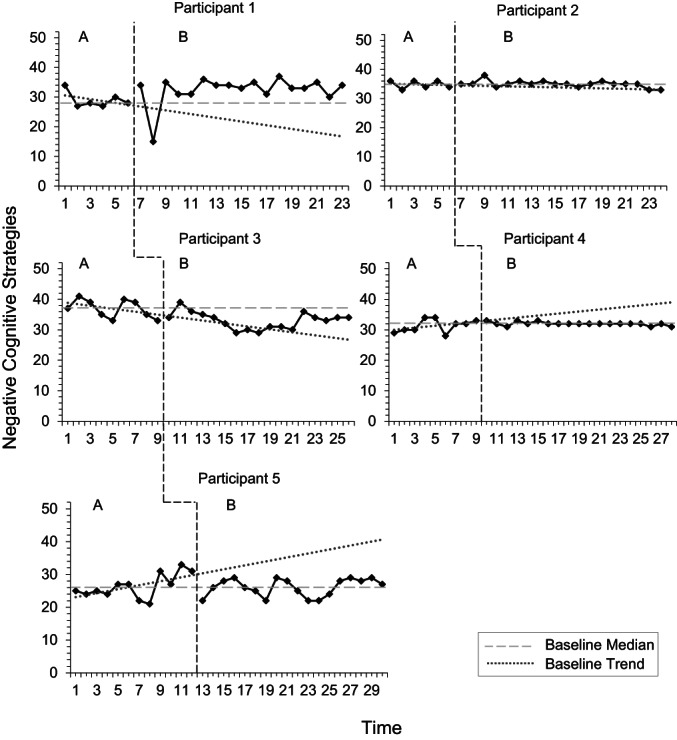
Use of negative cognitive strategies across baseline (A) and intervention (B) phases

Visual analysis of negative cognitive strategies (Figure 4) showed that these strategies did not reduce between baseline and intervention phases. Using the split‐middle method, the intervention phase only demonstrated a slight downward trend for P5. Analysing phase difference using PEM and DC methods illustrated no intervention effects on negative cognitive strategies (Table 11). This is consistent with Tau‐U calculations, according to which, no phase differences were evident across three of five participants. Despite this, P3 demonstrated a reduction in negative strategies, with moderate effects. Conversely, P1 demonstrated an increase in these strategies, with large effects. However, according to the overall weighted average Tau‐U calculation, no phase differences were evident.

#### Behavioural awareness and action

**FIGURE 5:**
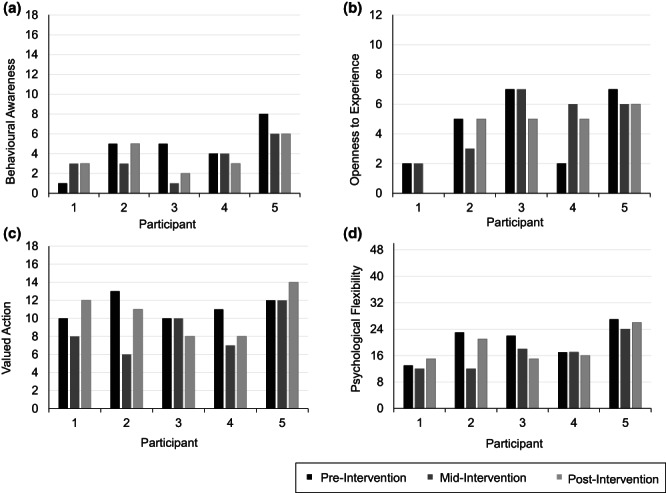
Participant scores on the CompACT‐8 at pre‐, mid‐, and post‐Intervention phases

The CompACT‐8 provided scores of behavioural awareness, openness to experience, valued action and psychological flexibility (Figure 5). Scores overall were lowest for behavioural awareness, and no clear trend was observed over time. Across all scales, scores appeared to be greatest for valued action; however, scores did not consistently increase over time. An increase in all scales was evident for P1, albeit remaining low throughout the study. Reductions across all scales were only evident for P3. Scores were largely stable for P5, and P2 demonstrated stability across scales when comparing pre‐intervention and post‐intervention scores. P4 evidenced variability in scores across all scales.

### Participant feedback

The Framework method was utilised to analyse written feedback provided by participants on an online survey. This systematic approach utilised a matrix output, enabling findings to be summarised according to theme. Three main themes were generated using a largely deductive approach, with an inductive approach utilised if additional themes were identified within the data. The main themes included (a) intervention, (b) change, (c) recommendations, outlined further in Table 12.

**TABLE 12:** Results from framework analysis: summary of themes from survey

Main theme	Subtheme	Key points	Examples
Intervention Factors	Acceptability	App overall acceptable for use	‘It was good, I would recommend it’
	Helpful aspects	Assisted participants in understanding selves	‘the reading in the guided journeys made me more educated on everything I go through’
		Enabled self‐monitoring of mood and health behaviours	‘From the app, I've been able to reflect and persevere. Just identifying feelings, situations and drawing lines and setting up boundaries in my head It allows me to judge situations I'm in and how I choose to react to them’
		Mindfulness content favoured by some	‘The meditations and videos have been really favourable parts of the app for me, they are simple yet engaging and are quite therapeutic to listen to’
	Hindering aspects	Difficult to find time to use app	‘it was very difficult to find the time and the right environment to do them’
		Difficult to motivate self to use app	‘The app is difficult to get motivated to do if you feeling down which is when you need to use the app most’.
		Some difficulties identifying and listing one's feelings	‘Listing my feelings was quite a difficult part overall’.
	Guidance	Weekly calls recognised as helpful and validating	‘I really relied on the weekly calls as she made me feel like all my thoughts, emotions and feelings were valid’
		Weekly calls sometimes disruptive to routine	‘My schedule was disrupted because there was more to handle than I initially realised’
Changes	Mood	Few changes in mood, some positive and some negative	‘my happy moods are happier, and my stressed moods are more stressful’
	Thoughts	Increase in use of positive strategies	‘I've learned that not everyone you meet on the street automatically dislikes you’
		Increase in thought challenging	‘I would always just go down a dark hole and started thinking about all the bad things I have experienced which would just make me feel extremely worse, but now when a situation happens I think about things that can uplift me and nice things to look forward to which makes me feel less depressed’.
	Behaviours	Increase in self‐regulation strategies	‘I've learned to try different techniques when feeling down or anxious, like deep breathing’
		Increase in use of mindfulness	‘Meditating helps me calm down if I am stressed’
		Decrease in avoidance behaviours	‘I always had negative coping strategies like just laying in bed to avoid everything but now I have more positive coping strategies like going for a walk and listening to music to help me calm down in the moment’
	External factors	Various additional factors influencing well‐being and ability to engage with app (e.g. COVID‐19 restrictions, observance of Ramadan, exams, broken device)	‘A lot happened during my time using the app that was out of both my and the apps control. Such as: my mum being hospitalised, exams, family issues, breaking my phone’
	Emotional insight	Greater insight into emotional experience, deemed both positive and negative by young people	‘It was also a bit of a shock as each week I would complete a new guided journey and it would highlight struggles that I face on a daily that I wasn't aware of or would push away’
Recommendations	App content	Content repetitive and time consuming; may be more appealing to condense the modules	‘The guided journeys are quite long and time consuming maybe they can be shorter but just as effective’
		Requirement for more advice within the app	‘information about what your family can do to help you’
	Technical	Smoother operation of features (e.g. not logging users out)	‘Make the app run smoother and not log out constantly’
		Enabling dyslexia‐friendly features	‘Maybe having an option to change the colour of the app maybe for people with dyslexia’
	Study design	Less repetition or change the order of questionnaires	‘repeating the questionnaires made me overanalyse my answers’
		Shorter calls via telephone rather than video platform	‘Having shorter calls and maybe over telephone’

## DISCUSSION

The primary aim of this study was to investigate evidence of change in psychological distress amongst adolescents while using a mental health app. This investigation did not find a replication of treatment effects across all five participants; the magnitude of change also depended on the method of analysis. According to the primary method of analysis, visual inspection of phase differences, treatment effects in reducing psychological distress were demonstrated across three participants, all of whom were recruited from the community sample. Despite this, only two participants demonstrated reductions in distress across all methods of analysis. Furthermore, a small overall effect of the app was found in reducing psychological distress; however, no improvements were evidenced in participants' well‐being.

The presented findings add to the mixed body of research investigating apps for adolescent mental health. For example, an RCT investigating the app ‘CopeSmart’ found no increases in well‐being, adaptive coping strategies or emotional self‐awareness, and no reductions in distress or dysfunctional coping strategies (Kenny et al., 2020). Additional trials amongst adolescents have reported no differences in depressive symptoms despite increases in well‐being and help‐seeking behaviours (O'Dea et al., 2020), opposing the presented results.

A range of possible reasons exist for the results. Firstly, it is acknowledged that the inferences drawn from the results depend on the analysis used. Visual analyses utilising DC and PEM methods were used to indicate phase changes in psychological distress. The findings according to this method suggest that amongst three participants within the community, app‐use demonstrated reductions in psychological distress. Supplemental analyses, however, highlight the possibility for results to vary depending on analytic method, which may lead to an under‐ or overstatement of treatment effects. However, the findings of the supplementary analyses within this study are to be interpreted cautiously, given the potential for bias with multiple testing (Šimundić, 2013).

A further explanation of the results is the possibility that participants did not actively engage with the content. Participant feedback highlighted hindering aspects of the app, including difficulties finding the time or motivation to use the app, even when recognising the potential benefits of engaging with the app content. Furthermore, inspection of usage patterns highlighted that large proportions of content were completed at once, on the day of, or days surrounding the weekly check‐ins. It is suggested that the rate of module completion may be greater for guided rather than unguided digital interventions (Baumeister et al., 2014), which may be due to an increased sense of accountability (Achilles et al., 2020). However, metrics of use do not equate to active engagement; thus, adherence rates may merely represent attendance, compliance or social desirability rather than meaningful interaction.

Another consideration for the present results considers app content. Given that Sanvello is based primarily upon CBT, the expected changes would be attributable to cognitive strategies. However, the findings here suggested that while participants exhibited an increase in adaptive strategies (such as positive reappraisal), changes were not evident in negative strategies (such as self‐blame). While skills in cognitive reappraisal facilitate regulation of negative affect, strategies such as rumination maintain or increase negative affect (Volkaert et al., 2019). Therefore, while this intervention demonstrated an overall moderate effect on adaptive cognitive strategies, this may not be sufficient in promoting positive clinical outcomes. This is consistent with cognitive theory, such that negative thinking styles should be relinquished to promote change (Beck, 1967). However, it is unclear why the intervention did not reduce maladaptive strategies.

As well as CBT, Sanvello utilises mindfulness; however, changes in psychological well‐being or psychological flexibility were not evident. While such result may be related to participant engagement, several participants reported subjective increased use of mindfulness following app use, suggesting potential preference for such content. As differences in mindfulness process variables did not appear to relate to participant feedback regarding mindfulness content, it is more likely that the results are due to the limited mindfulness content within this intervention. It would be hypothesised that behavioural awareness might have demonstrated greater change if the intervention was largely mindfulness or ACT‐based (Linardon, 2020).

A further explanation for the observed findings is provided by participants' feedback. Participants shared their experiences of gaining greater emotional insight, deemed as both positive and negative by different participants. Such subjective experience of insight might assist in understanding the different outcomes across participants. The role of emotional insight might have contributed positive change for some participants, consistent with a previous trial, in which adolescents using a self‐monitoring app demonstrated increased emotional self‐awareness (ESA) (Kauer et al., 2012; Reid et al., 2011). Within such trial, ESA was further associated with reduced depression and was proposed as a mediator of change. This is consistent with the suggestion that awareness is a prerequisite for emotion regulation (Lane & Schwartz, 1987). Despite the association between ESA and clinical outcomes within Kauer et al.'s (2012) research, a subjective increase in emotional insight in this study did not appear to covary with increased well‐being but might have contributed to the small changes in psychological distress.

It may be understood that rather than insight, subjective awareness of emotions and thoughts might instead elicit perseverative cognition or rumination (Volkaert et al., 2019; Watkins, 2008). However, in such case, deteriorating psychological outcomes would be expected. Within this investigation, it cannot be concluded that iatrogenic effects were evident. Despite P1 demonstrating an increase in distress, along with P2 exhibiting a reliable deterioration in well‐being, such results may be understood by considering individual context. For example, P2 acknowledged that towards latter stages of the intervention, observance of Ramadan led to fewer opportunities to engage with app content.

The possibility of iatrogenic effects should not however be dismissed. The results of this study in conjunction with existing literature do not demonstrate conclusive support for mental health apps. It may be possible that mobile devices are not conducive to therapeutic outcomes, and studies have begun highlighting the problematic nature of smartphone use. For example, adolescents exhibiting excessive mobile use also demonstrate more maladaptive cognitive strategies (Extremera et al., 2019). Accordingly, Mascia et al. (2020) found the relationship between self‐regulation strategies and well‐being was moderated by smartphone addiction. While smartphone addiction might have influenced the present results, this variable was not assessed, and such conclusions cannot be drawn. However, future investigations may explore smartphone addiction on the effectiveness of apps.

A notable finding here was participant attrition; despite two recruitment methods, 55% of participants withdrew. This is greater than the 47.8% dropout rate highlighted within a meta‐analysis exploring app studies (Torous, Lipschitz et al., 2020). Thus, despite some reductions in psychological distress, a greater proportion of adolescents were unable to engage with the intervention. This is consistent with feedback provided by participants, such that the app was time‐consuming and difficult to implement within their routines. Nonetheless, it is recognised that Sanvello has demonstrated high ratings of user experience (Mehdi et al., 2020), consistent with participants' favourable opinions of the app.

Without careful regulation, the potential harms of digital environments may be overlooked, for example potential addiction to devices, exposure of harmful content (Roland et al., 2020), lack of personalisation and difficulties with therapeutic relationships (Mind., 2021). At present, the Medicines and Healthcare Products Regulatory Agency (MHRA; 2021) guidelines concerning ‘medical devices’ do not include apps designed to monitor well‐being or provide education. As such, the applicability of these guidelines to mental health apps is ambiguous. Accordingly, while deemed ethically appropriate to use, the app utilised here was not registered with UK regulatory bodies. Given that current MHRA (2021) guidelines utilise terminology typifying the medical field, further regulation of psychologically informed interventions may be required. Such initiative would be important given the drivers to move towards digitally enabled care (NHS England, 2019; Topol, 2019).

The integration of digital technologies, rather than development of isolated tools such as apps, may instead be more beneficial to promote care in a flexible and responsive manner (Roland et al., 2020). Currently, NICE (2019) advocates the use of quality assured apps; however, guidelines and policies should be sensitive to the complex relationship between a multitude of variables when considering digital interventions for adolescents. Given that feedback provided by all participants illustrated the value of guidance, cost–benefit analyses of therapist guidance would aid the development of clinical guidelines and policy within the CYP‐IAPT model.

Despite the study's strength in utilising a multiple baseline design, several limitations are identified. First, this investigation lacked long‐term follow‐up, a consistent limitation across app studies (Badesha et al., 2022). Second, despite analysis of process variables, the ability to draw conclusions about causality is limited, given that factors beyond the control of the study design may not be identified. A range of external factors were identified by participants; however, there are likely further factors contributing to change, such as engagement. As active engagement remains a challenging variable to accurately measure (Achilles et al., 2020), the present results must be interpreted cautiously. Third, despite two recruitment methods, the sample observed a preponderance of community‐based participants, limiting the transferability of findings to clinical service contexts. A further limitation considers the measures utilised within this study. The K10 was administered three times per week, prompting for retrospection on the past week overall; however, it is not well‐validated for repeated measurement over short intervals. Accordingly, this might have conflated consecutive responses and limited sensitivity to change during both baseline and intervention phases. Finally, inclusion of variables such as ESA and smartphone addiction might have benefitted this investigation. However, further measures may enhance the possibility of a type I error (Field, 2013). Therefore, future research investigating the relationship between such variables would be fruitful in advancing the literature within this field.

## CONCLUSIONS

This study investigated evidence of change in psychological outcomes amongst five adolescents using a mental health app. The findings demonstrate that despite small reductions in psychological distress, the app did not lead to changes in psychological well‐being. Participants self‐reported subjective changes in their behaviour and thinking. Such findings were partially supported further, as participants evidenced increased adaptive cognitive strategies, but no changes in negative thinking strategies.

The large attrition rates suggest that there may be universal barriers to app engagement and adherence, in line with existing literature. Participants' feedback offered insights into the hindering aspects of the app, for example difficulties finding the time or motivation to engage with the content. Therefore, interpretation of results should consider such biases.

## AUTHOR CONTRIBUTION

**Dr Kiran Badesha:** contributed to conceptualization, investigation, methodology, recruitment, formal analyses, writing—original draft and writing—review and editing. **Dr Sarah Wilde:** contributed to conceptualization, methodology, supervision and Writing—review and editing. **Dr David L Dawson:** contributed to conceptualization, methodology, resources, supervision and writing—review and editing.

## CONFLICT OF INTEREST

The authors declare that there is no conflict of interest.

## Data Availability

The data that support the findings of this study are available from the corresponding author upon reasonable request.
